# Complete Genome Sequence of the Subsurface, Mesophilic Sulfate-Reducing Bacterium *Desulfovibrio aespoeensis* Aspo-2

**DOI:** 10.1128/genomeA.00509-14

**Published:** 2014-05-29

**Authors:** Karsten Pedersen, Andreas Bengtsson, Johanna Edlund, Lisa Rabe, Terry Hazen, Romy Chakraborty, Lynne Goodwin, Nicole Shapiro

**Affiliations:** a Microbial Analytics Sweden AB, Mölnlycke, Sweden; b Chalmers University of Technology, Gothenburg, Sweden; c Lawrence Berkeley National Laboratory, Berkeley, California, USA; d Department of Civil and Environmental Engineering, Earth and Planetary Sciences, Microbiology, University of Tennessee, Knoxville, Tennessee, USA; e Oak Ridge National Laboratory, Oak Ridge, Tennessee, USA; f Joint Genome Institute, Walnut Creek, California, USA

## Abstract

*Desulfovibrio aespoeensis* Aspo-2, DSM 10631^T^, is a mesophilic, hydrogenotrophic sulfate-reducing bacterium sampled from a 600-m-deep subsurface aquifer in hard rock under the island of Äspö in southeastern Sweden. We report the genome sequence of this bacterium, which is a 3,629,109-bp chromosome; plasmids were not found.

## GENOME ANNOUNCEMENT

*Desulfovibrio aespoeensis* was isolated from a borehole (KAS03) that intersected a water-conducting aquifer at 600-m depth (1). Knowledge about subsurface sulfate-reducing bacteria (SRB) is essential for evaluating adverse effects of their sulfide production on future spent nuclear fuel (SNF) waste repositories planned to be built 500 to 1,000 m underground. This is because metal canisters will be in use to encapsulate the SNF and many metals are susceptible to corrosion by sulfide (2). The safety case may consequently be challenged by subsurface SRB. Because *D. aespoeensis* readily reduces sulfate to sulfide with a very low *K*_*m*_ for hydrogen (3), this species and other hydrogenotropic SRB may increase anaerobic corrosion rates of iron in SNF repositories (4). It has previously been shown that *D. aespoeensis* forms biofilms of metallic copper (5), which indicates the presence of genes involved in copper homeostasis (6). Since the isolation, this species and closely related strains (>98% 16S rRNA gene identity) have been found repeatedly in deep groundwater, strongly suggesting the deep biosphere as the natural habitat for *D. aespoeensis* (7, 8).

The draft genome of *Desulfovibrio aespoeensis* Aspo-2 was generated at the U.S. Department of Energy (DOE) Joint Genome Institute (JGI) using a combination of Illumina and 454 technologies (project 4086336 [NCBI project 37869], principal investigator [PI] Terry C. Hazen). An Illumina GAii shotgun library was constructed which generated 36,973,545 reads totaling 1,331 Mb, a 454 Titanium standard library which generated 226,344 reads, and a paired-end 454 library with average insert sizes of 12 kbp which generated 58,898 reads totaling 208.3 Mb of 454 data. The initial draft assembly contained 73 contigs in 1 scaffold. The 454 Titanium standard data and the 454 paired-end data were assembled together with Newbler, version 2.3. Illumina sequencing data were assembled with VELVET, version 0.7.63.

The genome consists of one contig of 3,629,109 bp. The GC content is 62.6%, which agrees well with the previous melting point determination of 61 ± 0.5 mol% (1). Annotation predicted a total of 3,405 coding DNA sequences, as well as 39 pseudogenes, 2 rRNA genes, 53 tRNA genes, and 902 genes without identified functions.

Phylogenetic analysis based on completed genomes shows that the closest sequenced genomes to *D. aespoeensis* are those of *Desulfovibrio piezophilus* (9), *Desulfovibrio hydrothermalis* (10), and *Desulfovibrio salexigens*. These three species were isolated from marine habitats of which at least two are under considerable hydrostatic pressure (i.e., 26 and 17 MPa). *D. salexigens* was isolated from shallow sediments. Ji et al. (10) found that *D. aespoeensis* shares 2,069 orthologous proteins with *D. hydrothermalis* and 1974 proteins are common between these species and *D. salexigens*. Further analysis results by Ji et al. (10) suggest that *D. aespoeensis* has a phylogenetic position between marine and piezophilic species and nonmarine species such as *Desulfovibrio desulfuricans* and *Desulfovibrio alaskensis*. Comparative genomic analyses, including those of other *Desulfovibrio* species, will give insights into the adaptation of *D. aespoeensis* to subsurface habitats in hard rock aquifers and future SNF repositories.

### Nucleotide sequence accession number.

The complete annotated genome of *D. aespoeensis* is available in Genbank under the accession no. CP002431.1.
